# Dietary supplementation with *Chlorella vulgaris* in broiler chickens submitted to heat-stress: effects on growth performance and meat quality

**DOI:** 10.1016/j.psj.2024.103828

**Published:** 2024-05-04

**Authors:** M. Bošković Cabrol, A. Huerta, F. Bordignon, M. Pravato, M. Birolo, M. Petracci, G. Xiccato, A. Trocino

**Affiliations:** ⁎ Department of Agronomy, Food, Natural Resources, Animals and Environment (DAFNAE), University of Padova, Legnaro, Padova 35020, Italy; † Department of Comparative Biomedicine and Food Science (BCA), University of Padova, Legnaro, Padova 35020, Italy; ‡ Department of Agricultural and Food Sciences, Alma Mater Studiorum - University of Bologna, Cesena 47521, Italy

**Keywords:** microalgae, environmental temperature, myopathies, sex

## Abstract

Heat stress can greatly challenge growth and meat quality of broiler chickens where research is looking for sustainable ingredients, such as microalgae, that could also alleviate its negative impacts. Thus, in the present study, 576 1-D-old chicks (Ross 308) were housed until commercial slaughtering (42 D) in 36 pens in 2 rooms of a poultry house, according to a full factorial design encompassing 2 room temperatures (standard vs. high), 2 sexes (females vs. males), and 3 dietary treatments, that is, diet C0 (control diet), diet C3, and diet C6 containing 0, 3, and 6%, respectively, of *C. vulgaris* meal replacing the same quantities of soybean meal. The highest inclusion level of *C. vulgaris* decreased feed intake (*P* < 0.001) and body weight (*P* < 0.0001) compared to the control diet; it increased yellow and red indexes (*P* < 0.0001) of the breast muscle, besides the proportion of n3 polyunsaturated fatty acids (**PUFA**) (*P* = 0.028). Heat stress decreased feed intake (*P* = 0.001), breast (*P* = 0.001) and *p. major* yields (*P* = 0.036), and increased meat pH (*P*= 0.008) and cooking losses (*P* < 0.001), umami (*P* = 0.021) and brothy flavor (*P* < 0.001), and the proportion of n3 PUFA rates (*P* = 0.027), while reducing the contents of several amino acids in the breast meat (*P* ≤ 0.05). Compared to females, males displayed higher feed intake and growth, and more favorable feed conversion (*P* < 0.001). Carcass and *p. major* yields were greater in females (*P* < 0.001) which also showed a higher occurrence of spaghetti meat compared to males (*P* < 0.001). In conclusion, *C. vulgaris* can be used to replace until 3% of soybean meal in diets for broiler chickens without negative implications, while positively affecting breast meat color according to consumers’ preferences. However, the microalgae inclusion did not mitigate the negative effects of a chronic heat stress on growth performance nor reduced the occurrence of any myopathies.

## INTRODUCTION

Poultry meat is an important protein source worldwide as broiler chickens have a short production cycle and an efficient feed conversion ratio compared to other animals (Bromfield et al., 2021). In the European Union, poultry production increased by 3.2 million tons since 2007, reaching approximately 13.0 million tons of poultry meat in 2022 (Eurostat, 2023). Nevertheless, in light of global warming, conventional poultry production poses several challenges as for animals (EFSA, 2023) and environment (Zampiga et al., 2021a). As for animal welfare and health, commercial genotypes selected for high feed intake, fast growth rate and breast muscle hypertrophy are especially vulnerable to heat stress as they have fast metabolic activity and limited heat dissipation capacity (Uyanga et al., 2023). Available studies emphasized how acute and chronic stress results in poor performance and has detrimental effects on broiler health, welfare, and meat quality (Hu and Guo, 2008; El-Tarabany et al., 2021; Zampiga et al., 2021b). Thus, heat stress is a major environmental issue affecting poultry production worldwide (He et al., 2018; Zhang et al., 2020; Uyanga et al., 2023). Additionally, genetic selection has led to a high occurrence of growth-related myopathies, such as white striping (**WS**), wooden breast (**WB**) and spaghetti meat (**SM**) (Trocino et al., 2015; Petracci et al., 2019; Baldi et al., 2021), which has a big negative impact on meat quality and the poultry industry as a consequence of associated losses and wastes (Barbut, 2020; Bordignon et al., 2022; Che et al., 2022).

Then, as for environmental impacts of poultry production, feed has been recognized as a major contributor with special emphasis on the most currently used protein sources (Zampiga et al., 2021a). In this regard, microalgae have arisen as a promising alternative ingredient due to their high content of protein and essential amino acids, besides pigments and antioxidants, vitamins, n3 fatty acids, and minerals (Madeira et al., 2017; Alfaia et al., 2021). Along with spirulina (*Arthrospira platensis*), another unicellular freshwater microalga, *Chlorella vulgaris*, has been tested both as a supplement or ingredient in animal feeding (Madeira et al., 2017). In broiler chickens, previous studies found that low inclusion levels (0.015–1.0%) of *C. vulgaris* improved performance (An et al., 2016; Abdelnour et al., 2019; El-Bahr et al., 2020), whereas higher levels (10%) had positive effects on hepatic antioxidant capacity and meat quality (Alfaia et al., 2021; Coelho et al., 2021; Boskovic Cabrol et al., 2022a; Boskovic Cabrol et al., 2022b). Spirulina microalgae supplementation in water (5–20 g/L) (Kolluri et al., 2022) or feed (1–2 g/kg) (Elbaz et al., 2022; Abed et al., 2023) also improved performance, immunity, and antioxidant status in chickens under heat stress. Then, available studies about the impact of *C. vulgaris* on meat quality traits (El-Bahr et al., 2020; Alfaia et al., 2021; Boskovic Cabrol et al., 2022b) emphasized the positive effects on meat fortification with pigments and n3 polyunsaturated fatty acids (**PUFA**). On the other hand, there is an evident lack of information on if and how the microalgae inclusion in the diet, and specifically *C. vulgaris*, can affect the aforementioned muscles abnormalities, whereas minor effects of the dietary inclusion of spirulina on WB (Mullenix et al., 2022) and of docosahexaenoic acid (**DHA**)-rich microalgae on WS myopathies (Khan et al., 2021) have been reported. Being microalgae rich in bioactive compounds, we hypothesized that their dietary supplementation to broilers reared under chronic heat stress might improve growth performance and meat quality as well as decrease the occurrence of myopathies.

Thus, the present study aimed to evaluate the effect of the dietary inclusion and level of *C. vulgaris* (0, 3 and 6%) on growth performance, carcass yields, meat quality and nutritional value of breast meat, and growth-related myopathies occurrence in broiler chickens of both sexes kept under standard environmental conditions or submitted to a chronic heat stress.

## MATERIAL AND METHODS

### Ethical Statement

All procedures used in the present experiment were approved by the Ethical Committee for Animal Experimentation (*Organismo Preposto al Benessere Animale*) of the University of Padova (project number: 82/2022; Prot. n. 246564, 19/12/2022). All animals were handled in respect to the principles stated by the EC Directive 2010/63/EU regarding the protection of animals used for experimental and other scientific purposes. The researchers involved in animal handling were either animal specialists (PhD or MSc in Animal Sciences) and/or veterinary practitioners.

### Birds Housing, Management, and Diets

The trial was carried out in the poultry house of the Experimental Farm of the University of Padova (Legnaro, Padova, Italy) between the months of October and December, after a 6-month downtime. In the poultry house, 2 identical rooms were present both equipped with a cooling system, forced ventilation, radiant heating, and controlled light systems. A total of 576 one-day-old chicks of both sexes (Ross 308, Aviagen Group, NW Huntsville, AL) were transported from a commercial hatchery by an authorized truck to the experimental facilities in compliance with Council Regulation (EC) No 1/2005. Chicks had been sexed and vaccinated against Marek's disease, Infectious Bronchitis, and Newcastle disease at the hatchery. At their arrival, chicks were individually weighed, identified by a leg mark, and randomly allocated among the 36 pens (16 chickens per pen) in the 2 rooms of the poultry house (18 pens per room) which were managed differently for temperature to maintain standard temperature (**ST**) conditions in the first room and to submit chickens to a moderate heat stress in the adjacent high-temperature room (**HT**), as detailed below. Thus, the 36 pens were allocated to 12 experimental groups, that is, 2 temperatures (ST room and HT room) × 2 sexes (males and females) × 3 diets with different inclusion level of *C. vulgaris* (0, 3, and 6%). Then, 504 broiler chickens were used for the purposes of the present study, whereas the other 72 chickens were slaughtered at 2 different ages (13 D and 32 D) for sampling muscle tissues, gut mucosa, and gut microbiota (data not reported in the present paper). Each pen (2.2 m^2^; 125 cm wide × 177 cm long × 120 cm height) had a circular feeder (diameter: 37 cm) for the manual distribution of the diet and a drinker line with 5 nipples. The concrete floor was covered with wood shavings litter (height 5 cm, 2.5 kg/m^2^). Light was provided for 24 h/D on the first day after arrival supplementing natural light with artificial light (light source: Osram L 36W/640 cool white; OSRAM Licht AG, Munich, Germany). From the second day of age, the hours of lightness progressively decreased until an 18 light:6 dark photoperiod was achieved which was then maintained from 12 D of age onwards.

The 2 twin rooms of the poultry house were differently managed for temperature, in order to maintain standard temperature (**ST**) conditions as recommended for broiler chickens (Aviagen, 2018) in the first room and to submit chickens to a moderate heat stress in the adjacent high-temperature room (**HT**). In details, temperature was kept at 31 ± 1°C during the first 3 d and then gradually reduced by 2°C per week until reaching 20°C by 35 d of age in the ST room. In the HT room, the temperature was 31±1°C, 30 ± 0.5°C, 29± 0.5°C and 28± 0.5°C during the periods 1 to 6 d, 7 to 13 d, 14 to 20 d, and 21 to 42 d of age, respectively. Data loggers (P5185, PeakTech, Prüfund Messtechnik GmbH Gerstenstieg, Ahrensburg, Germany) were used to record temperature and relative humidity.

The feeding plan during the trial used different basal diets according to the growth period, that is, a starter diet (mash form) from 0 to 13 D of age; a grower diet (mash form) from 14 to 27 D; and a finisher diet (crumble form) from 28 to 42 D, to meet broiler nutritional requirements (Table 1). During the first 5 d upon arrival, all chickens were fed the control diet (diet C0), made up by 94% basal diet and 6% soybean meal (Table 1). Then, from 6 D of age until commercial slaughtering at 42 D of age, chickens received one of the following 3 dietary treatments (Table 1) during the starter, grower, and finisher periods: 1) the diet C0; 2) the diet C3, made up by 94% basal diet of the corresponding period, 3% soybean meal, and 3% *C. vulgaris*; or 3) the diet C6, made up by 94% basal diet of the corresponding period and 6% *C. vulgaris*. The basal diets were produced by a commercial feed mill (Consorzio Agrario di Treviso e Belluno, Paese, Treviso, Italy). The *C. vulgaris* meal was obtained from ALLMICROALGAE - Natural Products SA (Pataias, Portugal). The experimental diets (C0, C3, and C6) were obtained at the experimental farm mixing the basal diets with the due quantities of soybean meal and *C. vulgaris*. The chemical, mineral, and fatty acid composition of the basal diets, soybean meal, and *C. vulgaris* are presented in Table 2, Table 3. Throughout the experiment, birds were offered feed and water *ad libitum*.

**Table 1:** Ingredients (g/kg as fed) of the basal diets.

	Basal diets		
	Starter	Grower	Finisher
Corn meal	499.0	536.3	559.0
Soybean meal (CP 48%)	297.0	200.0	180.0
Corn gluten feed	59.0	79.0	79.0
Wheat middlings	50.0	70.0	70.0
Full fat soybean	0.0	25.0	25.0
Wheat bran	19.0	18.0	20.0
Soybean oil	17.0	17.0	16.8
Monocalcium phosphate	17.0	16.6	10.0
Calcium carbonate	16.6	15.0	17.7
Sunflower meal (CP 28%)	10.0	10.0	10.0
Sodium chloride	2.0	2.0	2.0
Sodium bicarbonate	2.0	2.0	2.0
L-lysine HCl	1.8	1.4	1.1
DL-methionine	1.4	1.4	1.1
L-threonine	0.5	0.3	0.3
Liquid coline 70%	0.4	0.4	0.4
Mineral and vitamin premix^1^	7.3	5.6	5.6

CP: crude protein.

1 Premix ingredients (per kg premix): vit. A, 1,600,000 IU; vit. D3, 500,000 IU; vit. E, 5,000 mg; iron sulphate (Fe): 7,000 mg; calcium iodate anhydrous (I): 100 mg; cupric sulphate (Cu): 1,500 mg; manganese oxide (Mn): 21,000 mg; zinc oxide (Zn): 8400 mg; sodium selenite (Se): 60 mg; 6-phytase (EC 3.1.3.26): 120,000 OTU; Endo-1,4-beta-xilanasi EC 3.2.1.8 (4a7), 112000 TXU; Endo-1,4-beta-glucanasi EC 3.2.1.4 (4a7), 50,000 TGU.

**Table 2:** Chemical composition (% as fed) and mineral content (mg/kg) of the experimental diets and the raw materials (soybean meal and *Chlorella vulgaris*).

	Experimental diets										
	Starter			Grower			Finisher			Raw materials	
Composition	C0	C3	C6	C0	C3	C6	C0	C3	C6	Soybean meal	*C. vulgaris*
Dry matter	89.9	90.3	90.5	88.3	88.4	88.5	89.1	89.1	89.4	89.7	94.4
Crude protein	21.0	20.2	21.2	18.3	18.8	19.1	17.8	19.0	19.3	44.3	49.2
Ether extract	3.79	4.02	3.97	3.87	3.98	4.18	4.44	4.66	4.61	1.16	0.81
Starch	32.8	32.9	35.2	34.4	35.7	36.1	38.0	35.8	36.8	3.64	8.61
Ash	6.65	6.57	6.73	5.91	6.13	6.34	6.39	6.09	6.57	6.71	7.49
Na	1912	1795	1789	816	2722	876	1775	1736	1872	332	2346
K	10983	9796	10070	9920	9248	8682	9791	9360	9399	21355	8835
Ca	13057	11050	11338	9600	10218	10853	11475	12960	13097	3508	11958
Mg	2289	2139	2190	2000	1909	1853	1905	1936	1957	3115	2186
P	8295	8072	8662	7432	7607	7907	7168	8080	8089	7025	17975
Fe	259	334	311	194	225	260	264	275	304	170	1112
Cu	13.0	10.8	12.2	7.19	13.2	8.14	16.8	13.8	11.3	14.1	18.7
Zn	89.1	94.8	91.5	93.6	94.0	93.8	76.1	80.0	91.7	49.4	268
Mn	120	122	116	126	122	129	150	141	151	46.6	101
S	2381	2484	2599	2200	2364	2411	2433	2552	2712	3821	7388

C0: Control diet (94% basal diet + 6% soybean meal); C3: 94% basal diet + 3% soybean meal + 3% *C. vulgaris*; C6: 94% basal diet + 6% *C. vulgaris*.

**Table 3:** Fatty acid profile (g/100 g of total fatty acids) of the experimental diets.

	Experimental diets								
	Starter			Grower			Finisher		
Fatty acids	C0	C3	C6	C0	C3	C6	C0	C3	C6
C16:0	12.1	12.4	12.3	12.0	12.3	12.5	11.7	11.8	11.8
C18:0	3.28	3.62	3.55	3.35	3.43	3.51	3.43	3.26	3.54
C20:0	0.41	0.46	0.44	0.40	0.42	0.45	0.42	0.41	0.47
Other SFA	0.94	0.91	1.00	1.01	1.01	0.93	0.90	0.90	0.90
C18:1n9	24.9	25.8	25.6	25.6	25.5	25.9	25.3	25.0	25.6
C18:1n7	1.09	1.11	1.11	1.06	1.09	1.11	1.09	1.09	1.10
Other MUFA	0.62	0.65	0.69	0.68	0.71	0.75	0.54	0.62	0.68
C18:2n6	52.2	50.7	51.0	51.8	51.3	50.7	51.7	51.9	51.0
C18:3n3	4.33	4.21	4.16	3.96	4.14	4.01	4.86	4.97	4.84
Other PUFA	0.13	0.12	0.13	0.13	0.11	0.13	0.08	0.08	0.10
Total SFA	16.7	17.4	17.3	16.8	17.1	17.3	16.4	16.4	16.7
Total MUFA	26.6	27.6	27.4	27.3	27.3	27.8	26.9	26.7	27.4
Total PUFA	56.6	55.1	55.3	55.9	55.5	54.9	56.6	56.9	55.9
n6	52.3	50.8	51.2	51.9	51.4	50.8	51.8	52.0	51.1
n3	4.33	4.21	4.16	3.96	4.14	4.01	4.86	4.97	4.85
n6/n3	12.1	12.1	12.3	13.0	12.4	12.7	10.6	10.5	10.5

SFA: saturated fatty acids; MUFA: monounsaturated fatty acids; PUFA: polyunsaturated fatty acids. C0: Control diet (94% basal diet + 6% soybean meal); C3: 94% basal diet + 3% soybean meal + 3% *C. vulgaris*; C6: 94% basal diet + 6% *C. vulgaris*.

Chickens were weighed on their arrival and then once per week whereas pen feed intake was recorded daily. Mortality and health conditions were daily checked. During the trial, 5 chickens died and 4 chickens were excluded from the trial due to leg problems.

### Slaughtering, Carcass and Meat Quality Traits

At 42 D of age, all remaining chickens were individually weighed before crating after 4-h fasting. Then, they were placed into a transport cage (height, 62.5 cm × 160 cm × 25.0 cm; floor area, 1 m^2^), transported for approximately 15 min to a commercial poultry processing plant and slaughtered following the standard commercial procedures. Carcass weights were recorded after 2 h of refrigeration at 2 °C to calculate carcass yield. Then, 180 carcasses (5 per pen) were selected as representative in terms of average live weight and variability of the corresponding pens and submitted to gross examination of *pectoralis major* for the occurrence and severity of white striping and wooden breast (Kuttappan et al. 2012; Sihvo et al., 2014), and the occurrence of spaghetti meat (Baldi et al., 2021). Afterwards, 108 carcasses (3 per pen) were transported to the DAFNAE laboratories and stored in a refrigerated room at 4 °C. Twenty-four hours *post mortem*, carcasses were cut in major parts (breast, wings, and drumsticks with thighs) and *p. major* muscles were separated from the breasts. The right *p. major* muscle was immediately used for measuring pH and color and then processed for chemical analyses of meat, as detailed below. The left muscles were vacuum packaged, stored at –18 °C and later used for measuring thawing and cooking losses, texture, and sensory characteristics on a total of 72 breasts (2 per pen; 36 per temperature, 36 per sex, 24 per diet).

As for meat rheological traits, in details, the pH of breast meat was determined in triplicate on their ventral side using a pH meter (Basic 20, Crison Instruments Sa, Carpi, Italy) equipped with a specific electrode (cat. 5232, Crison Instruments Sa). The instrumental color indices were measured on the same positions after approximately 30 min of blooming at room temperature using a Minolta CM-508 C spectrophotometer (Minolta Corp., Ramsey, NJ), with illuminant 65D. Then, Texture Profile Analysis (TPA) was performed using a TA.HDI dynamometer (Stable Micro System Ltd., Goldaming, UK), with a 20 mm-diameter cylindrical probe, moving with 5 mm compression (up to 40% of original sample height) at a constant speed of 2 mm/s for 2 consecutive cycles, separated by a 5-s interval. The Texture Export software (Stable Micro System Ltd.) was used to calculate hardness (N), springiness (mm), cohesiveness, and chewiness (N × mm). Hardness was the peak force required for the first compression; cohesiveness was the ratio of active work done under the second compression curve to that done under the first compression curve; springiness was the tissue response to the first compression to return to the pre-compressive status (ratio between the first work area and the second work area); and chewiness was the product hardness × cohesiveness × springiness.

Thawing and cooking losses were determined according to Petracci and Baéza (2011). The shear force was measured on cooked meat using LS5 dynamometer (Lloyd Instruments Ltd, Bognor Regis, UK) with the Allo-Kramer (10 blades) probe (load cell: 500 kg; distance between the blades: 5 mm; thickness: 2 mm; cutting speed: 250 mm/min) (Mudalal et al., 2015; Gratta et al., 2023).

### Meat Chemical Composition

Breast meat (from 2 carcasses per pen; 72 in total; 36 per temperature, 36 per sex, 24 per diet) was freeze-dried, re-ground, and used to determine proximate composition, that is, dry matter (934.01), ash (967.05), crude protein (2001.11), and ether extract (991.36) contents (AOAC, 2000), besides fatty acid composition, mineral and amino acid content, as detailed below.

As for fatty acid (**FA**) profile, fat was extracted from freeze-dried meat by accelerated solvent extraction (ASE, Dionex, Sunnyvale, CA, Application Note 334) using 3 extraction cycles with chloroform:methanol (2:1 v/v) as a solvent at 80 °C and a 1-min heating phase and 40-s extraction phase. The solvent was evaporated under a N_2_ stream (Genevac EZ-2, SP Industries, Warminster, PA) at 60 °C; the residual samples (extracted lipids in vials) were weighed before adding 4 mL of 1% H_2_SO_4_ in methanol and held at 50 °C overnight. Then, hexane (1 mL hexane for 20 mg extracted fat) and 4 mL of NaSO_4_ (0.47% in H_2_O) were added and vigorously agitated to transfer the methylated fatty acids in the organic phase. The organic phase was collected after centrifugation and analyzed by GC-FID with an 7820A Gas Chromatograph (Agilent Technologies, Santa Clara, CA). A total of 1 μL was injected with a split ratio of 65:1. A Supelco OMEGAWAX-TM 250 (Sigma-Aldrich, St. Louis, MO) (30 m × 0.25 mm internal diameter, 0.25 μm film thickness) was used with hydrogen as the carrier at 1.4 mL/min. The oven temperature was set at 50 °C, held for 2 min, raised to 220 °C at the rate of 4 °C/min, and then held for 23 min. Both the injector and the detector temperatures were set at 250 °C. The individual FA were identified by comparing the retention time of the standard FA methyl esters mixture (Supelco 37–component FAME Mix, 47,885–U). Individual FA methyl esters were expressed as the percentage of the total area of eluted FA methyl esters.

As for mineral content, 0.30 to 0.35 g of freeze-dried meat from 36 breasts (1 per pen; 18 per temperature, 18 per sex, 12 per diet) were weighed and placed in a tetra-fluorine modified (**TFM**) vessel with 2 mL of 30% Suprapur hydrogen peroxide and 7 mL of concentrated (65%) Suprapur nitric acid (Merck Chemicals GmbH, Darmstadt, Germany). The samples were subjected to microwave digestion (Ethos 1600 Milestone S.r.l., Sorisole, Bergamo, Italy) according to the following steps: 1) 25 to 200 °C during 15 min at 1,200 W with P max 100 bar; 2) 200 °C during 18 min at 1200 W with P max 100 bar; and 3) 200 to 35 °C in 15 min. After cooling to room temperature, the dissolved sample was diluted with ultrapure water (resistivity 18.2 M Ω cm at 2 5°C) to a final volume of 25 mL. The mineral contents were determined with a Spectro Arcos EOP inductively coupled plasma–optical emission spectrometry (ICP-OES; Spectro Analytical Instruments GmbH, Kleve, Germany). Calibration standards were prepared using multi- and single-element standard solutions (Inorganic Ventures Inc., Christiansburg, VA) in 30% Suprapur nitric acid (Merck Chemicals GmbH) to obtain similar matrices to the samples.

Amino acids were determined after acid hydrolysis and pre-column derivatization with 6-aminoquinolyl-N-hydroxysuccinimidyl carbamate, separated by RP-HPLC and analyzed by UV detection following a method adapted from European Pharmacopoeia. Briefly, for alanine, arginine, aspartatic acid, glutamic acid, glycine, histidine, isoleucine, leucine, lysine, methionine, phenylalanine, proline, serine, threonine, tyrosine, and valine determination, protein of the sample was hydrolyzed with hydrochloride acid (6 M) at 105 °C for 24 h. Cysteine (sum of cystine and cysteine) was determined after reaction with dithiodipropionic acid, producing a mixed disulphide, which then underwent acid hydrolysis. After hydrolysis, the samples were neutralized with sodium hydroxide (8 M), adjusted to volume and filtered at 0.22 µm. Then, the derivatization step was conducted according to the manufacturer's instructions (AccQTag Ultra Derivatization Kit, Waters Corporation, Milford, MA). Tryptophan was determined following a method adapted from Commission Directive 2000/45/EC (EC, 2000) using a basic hydrolysis with barium hydroxide at 105 °C for 24 h, and after neutralization and filtration analyzed directly by RP-HPLC. Separation and quantification of amino acids were performed using an Agilent 1,260 Infinity HPLC (Agilent Technologies) equipped with a reversed-phase column C18 (CORTECS C18, 2.7 µm, 2.1 × 150 mm, Waters Corporation) kept at 45 °C, and with a diode array Detector (1260 Series, DAD VL+, Agilent Technologies).

### Meat Oxidation

Protein carbonylation in breast meat was determined according to 2,4-dinitrophenylhydrazine (**DNPH**) -based method, as described by Levine et al. (1990) and modified by Soglia et al. (2016). One gram of sample was homogenized with 10 mL of ice-cold 0.15 M KCl solution using an Ultra-Turrax T25 basic (IKA – WERKE, Labortechnik, Staufen, Germany) homogenizer at 9500 rpm for 30 s. Then, 4 aliquots (100 μL) of the homogenate were mixed with 1 mL of 10% trichloroacetic acid (**TCA**) and centrifuged at 5,000 × *g* for 5 min (Centrifuge 5424, Eppendorf AG, Hamburg, Germany). Supernatant was discarded and 400 μL of 5% sodium dodecyl sulfate were added to the pellet. The mixture was heated at 100 °C for 10 min and ultrasonicated (Argo-lab DU-45, Modena, Italy) at 40 °C for 30 min, after which the samples (3 replicates) were treated with 0.8 mL of 0.3% (w/v) DNPH in 3 M HCl while the same volume of 3 M HCl were added to the blank. After 30-min incubation at room temperature, 400 μL of 40% TCA were added to precipitate the proteins and samples were centrifugated at 5,000 × *g* for 5 min. Then, supernatant was removed and the pellet was washed 3 times with 1 ml of ethanol–ethyl acetate (1:1, v:v) solution by centrifugation at 10,000 × *g* for 5 min. After the final wash, the pellets were dried at room temperature for 1 h, dissolved in 1.5 mL of 6 M guanidine hydrochloride in 20 mM NaH_2_PO_4_ (pH 6.5), and placed at 4 °C overnight. After incubation, the transmittance of the samples was measured spectrophotometrically (UV–VIS 1800 spectrophotometer, Shimadzu Corporation, Kyoto, Japan) at 280 nm and 370 nm and carbonyl content, expressed as nmol/mg of protein, was calculated according to the following equation:

$$\text{Carbonyl}\;\text{content}\;\left[\text{nmol}/\text{mg}\;\text{protein}\right]=\frac{\text{Abs}370-\text{Abs}370\;\left(\text{blank}\right)}{22,000\times [\text{Abs}280-\left(\text{Abs}370-\text{Abs}370\;\left(\text{blank}\right)\times 0.43\right]}\times 10^{6}$$

Lipid oxidation was determined by the thiobarbituric acid reactive substances (**TBARS**) assay using the method by Bao and Ertbjerg (2015) with slight modifications. A total of 5 g of sample (in triplicate) were homogenized in 15 mL TCA (5%, w/v) and 0.5 ml butylated hydroxytoluene (4.2% in ethanol, w/v) using Ultra-Turrax (IKA, Labortechnik, Staufen, Germany). Then, the homogenate was filtered (Whatman 1, GE Healthcare, Chicago, IL) and the mixture of 2 mL of filtrate and 2 mL thiobarbituric acid (0.02 M) was placed in a boiling water bath (100 °C) for 40 min. After cooling samples with running tap water to room temperature, the absorbance was measured spectrophotometrically at 530 nm (UV–VIS 1800 spectrophotometer, Shimadzu Corporation). The TBARS content, expressed as mg malondialdehyde (MDA)/kg of meat, were calculated from a standard curve generated with 1,1,3,3-tetraethoxypropane.

### Meat Sensory Evaluation

Quantitative descriptive sensory analysis (**QDA**) of the *p. major* muscle was carried out by 12 panelists (7 females and 5 males, aged 27–55 yr) with 3-yr sensory evaluation experience, recruited from the department DAFNAE and trained according to ISO standards (ISO 8586, 3972, 5496). The panelists were trained to ensure the competent usage of sensory descriptors. Then, 72 breasts (6 per 12 experimental groups) were randomly evaluated across 7 sessions during 4 wk. In each session, assessors evaluated 2 sets of 3 chicken breasts, with 15-min break between sets. The evaluated attributes were color, odor, taste, texture, and overall acceptability. Each attribute was evaluated on a 15 cm structured continuous line scale with anchor points at 0 (not intense) and 10 (very intense).

After thawing at 4 °C for 24 h, meat samples were individually cooked in sealed vacuum bags in a water bath set at 85 °C in plastic bags until reaching an internal temperature of 78 °C. Then, each meat piece was divided into 4 equal samples (3 cm × 2 cm × 1 cm) and immediately served to the panelists. The samples were coded with 3-digit codes and presented in white plastic cups. Sample presentation order was systematically varied using a Williams Latin square design to balance the effects of serving order and carryover effect. After each sample, panelists cleaned their palate with a piece of apple, an unsalted cracker, and mineral water. Data were collected using the Fizz v2.47b software program (Biosystems, Couternon, France).

### Statistical Analyses

Individual data of live weight, daily weight gain, and carcass and meat quality traits were submitted to the analysis of variance (**ANOVA**) using the PROC MIXED of SAS software (SAS, 2013), with diet, environmental temperature, and sex, as main factors of variability with interactions, and the pen as a random effect, as follows:

$$Y_{ijkl}=\mu +D_{i}+T_{j}+S_{k}+{DT}_{ij}+{DS}_{ik}+{TS}_{jk}+{DTS}_{ijk}+e_{ijkl}$$

where µ is an overall mean response, D represents the effect of the _i_ level of the Diet (0, 3, 6% *C. vulgaris*), T represents the effect of the _j_ level of the environmental Temperature (standard vs. heat stress), S represents the effect of the _k_ level of the Sex (females vs. males), Y is the response of the experimental unit, and e is the observation error.

Pen data of feed intake and feed conversion ratio were submitted to ANOVA considering the same main factors and using the PROC GLM (SAS, 2013), whereas the occurrence of myopathies was analyzed with the PROC CATMOD of SAS. Results of sensory meat analyses were processed using the PROC MIXED of SAS with a model including diet, environmental temperature, and sex, as main factors of variability with interactions and the panelist as a random effect. Differences among the means with *P* ≤ 0.05 were accepted as statistically significant.

## RESULTS

### Growth Performance and Carcass Traits

The dietary inclusion of *C. vulgaris* significantly impaired growth performance at the highest inclusion level, that is, 6%, which decreased final body weight, daily weight gain, and feed intake during the starter, grower, and finisher periods and on the whole trial compared to chickens fed the control diet and the diet containing 3% *C. vulgaris* (Table 4). Feed conversion ratio (**FCR**) during the starter period was better in chickens fed the diet containing 6% *C. vulgaris* compared to chickens fed the control diet (1.27 vs. 1.31; *P* = 0.01), which did not affect FCR during the whole trial being similar among dietary treatments. At slaughtering, breast, and *p. major* yields were significantly greater in chickens fed the highest level of microalgae inclusion compared to those of the control diet, whereas those fed 3% of *C. vulgaris* showed intermediate values (Table 5). On the other hand, the occurrence of wooden breast tended to be lower in chickens fed the highest level of *C. vulgaris* (11.3%, 11.7%, and 1.67% in chickens fed diets C0, C3, and C6, respectively; *P* = 0.05) (Table 5).

**Table 4:** Productive performance^1^ (LS means) of broiler chickens fed diets with different inclusion levels of *Chlorella vulgaris* and reared under standard or high room temperature in separated sexes from hatching until commercial slaughtering.

	Experimental diet (D)			Room temperature (T)		Sex (S)		*P* value							RMSE
Items	C0	C3	C6	Standard	High	Female	Male	D	T	S	D×T	D×S	T×S	D×T×S	
Chickens (n)	163	166	161	243	247	247	243								
Body weight (g)															
1 D	45.7	45.9	45.2	45.2	46.0	44.6	46.7	0.192	0.206	<0.001	0.502	0.596	0.758	0.689	3.5
14 D	438^a^	454^b^	438^a^	443	443	426	460	<0.001	0.849	<0.001	0.560	0.616	0.882	0.093	45.7
27 D	1,170^a^	1,196^a^	1,155^b^	1,183	1,165	1,121	1,228	<0.001	0.298	<0.001	0.787	0.088	0.420	0.820	114
42 D	2,516^ab^	2,564^a^	2,484^b^	2,569	2,474	2,310	2,737	<0.001	0.058	<0.001	0.371	0.041	0.596	0.749	191
Starter period (1-14 D)															
Weight gain (g/D)	28.0^a^	29.1^b^	28.0^a^	28.4	28.3	27.3	29.5	<0.001	0.686	<0.001	0.586	0.570	0.801	0.090	3.2
Feed intake (g/D)	36.6^a^	37.3^a^	35.6^b^	36.7	36.3	35.5	37.4	<0.001	0.170	<0.001	0.162	0.723	0.232	0.056	0.9
Feed conversion (g/g)	1.31^a^	1.28^ab^	1.27^b^	1.29	1.28	1.30	1.27	0.010	0.099	0.001	0.570	0.298	0.156	0.942	0.02
Grower period (15-27 D)															
Weight gain (g/D)	56.3^a^	57.1^a^	55.2^b^	56.9	55.6	53.5	59.0	<0.01	0.094	<0.001	0.777	0.098	0.383	0.632	7.5
Feed intake (g/D)	90.9^ab^	93.1^a^	88.7^b^	92.1	89.8	88.1	93.7	0.032	0.082	<0.001	0.281	0.374	0.379	0.521	3.8
Feed conversion (g/g)	1.62	1.63	1.62	1.63	1.62	1.65	1.60	0.798	0.788	0.038	0.281	0.396	0.955	0.320	0.07
Finisher period (28-42 D)															
Weight gain (g/D)	89.7^ab^	91.2^a^	88.6^b^	92.4	87.3	79.2	101	0.001	<0.001	<0.001	0.286	0.087	0.935	0.408	8.1
Feed intake (g/D)	173^a^	172^a^	164^b^	172	167	157	182	<0.001	<0.01	<0.001	0.026	0.014	0.734	0.004	4.6
Feed conversion (g/g)	1.94	1.90	1.88	1.88	1.93	1.98	1.92	0.253	0.093	<0.001	0.162	0.586	0.672	0.224	0.09
Whole trial (1-42 D)															
Weight gain (g/D)	58.8^ab^	59.9^a^	58.1^b^	60.1	57.8	53.9	64.1	<0.001	0.060	<0.001	0.380	0.040	0.592	0.748	4.5
Feed intake (g/D)	104^a^	104^a^	99.4^b^	104	101	96.5	108	<0.001	0.001	<0.001	0.164	0.015	0.389	0.010	2.3
Feed conversion (g/g)	1.76	1.75	1.73	1.74	1.75	1.79	1.70	0.268	0.321	<0.001	0.189	0.386	0.928	0.139	0.05

RSME, root mean square error. C0: Control diet (94% basal diet + 6% soybean meal); C3: 94% basal diet + 3% soybean meal + 3% *C. vulgaris*; C6: 94% basal diet + 6% *C. vulgaris*.

1 Individual data: live weight and daily growth rate. Pen data: feed intake and feed conversion.

a,b Different superscripts within a row indicate a significant difference (*P* ≤ 0.05).

**Table 5:** Carcass traits (LS means) and growth-related myopathy occurrence at 42 D of age in broiler chickens fed diets with different inclusion levels of *Chlorella vulgaris* and reared under standard or high room temperature in separated sexes until commercial slaughtering.

	Experimental diet (D)			Room temperature (T)		Sex (S)		*P* value							RMSE
	C0	C3	C6	Standard	High	Female	Male	D	T	S	D×T	D×S	T×S	D×T×S	
Chickens (n)	36	36	36	54	54	54	54								
Cold carcass weight (g)	1812	1830	1790	1825	1796	1681	1940	0.197	0.108	<0.001	0.945	0.044	0.272	0.693	93
Carcass yield (%)	72.9	72.5	73.0	72.8	72.8	73.6	72.0	0.495	0.987	<0.001	0.277	0.380	0.203	0.700	1.8
Breast yield (%)	38.2^a^	39.2^ab^	39.6^b^	39.7	38.4	39.9	38.1	<0.01	0.001	<0.001	0.447	0.160	0.719	0.881	1.9
*P. major* muscles (%)	24.1^a^	25.0^ab^	25.3^b^	25.1	24.5	25.5	24.0	<0.01	0.036	<0.001	0.253	0.021	0.714	0.469	1.6
Wings (%)	9.99	10.0	10.0	9.99	10.1	10.0	10.0	0.881	0.411	0.886	0.370	0.370	0.370	0.370	0.49
Legs (%)	31.1	30.6	30.1	29.8	31.5	29.9	31.3	0.077	<0.001	<0.001	0.277	0.561	0.157	0.157	1.9
Chickens (n)	60	60	60	90	90	90	90								-
White striping (%)	21.7	20.0	35.0	24.4	26.7	26.7	24.4	0.119	0.732	0.732	0.071	0.498	0.059		-
Wooden breast (%)	13.3	11.7	1.67	7.78	10.0	5.56	12.2	0.052	0.600	0.116	0.960	0.718	0.739		-
Spaghetti meat (%)	16.7	23.3	15.0	16.7	20.0	28.9	7.78	0.458	0.563	<0.001	n.e.	n.e.	n.e		-

RSME: root mean square error. C0: Control diet (94% basal diet + 6% soybean meal); C3: 94% basal diet + 3% soybean meal + 3% *C. vulgaris*; C6: 94% basal diet + 6% *C. vulgaris*. n.e.: not estimable.

a,b,c Different superscripts within a row indicate a significant difference (*P* ≤ 0.05).

As for room temperature, weight gain (+5.80%; *P* < 0.001) and feed intake (+3.0%; *P* < 0.01) were higher in chickens kept under standard conditions compared to those kept under chronic heat stress during the finisher period. Thus, in the whole trial, feed intake (*P* = 0.001) was higher in chickens kept under standard conditions compared to those kept under heat stress, whereas differences in daily weight gain only showed a trend (*P* = 0.060) and feed conversion ratio was not affected (Table 4). Then, breast and *p. major* yields were greater (0.01 < *P* ≤ 0.05) and leg proportion lower (*P* < 0.001) in chickens kept under standard conditions compared to those kept at high temperature (Table 5).

Finally, as expected, compared to females, males displayed significantly (*P* < 0.001) higher body weight, daily weight gain, and feed intake, which corresponded to a more favorable feed conversion ratio since the beginning of the trial and during the whole experimental period (Table 4). At slaughtering, carcass, breast and *p. major* yields were greater in females compared to males (*P* < 0.001), whereas at gross examination the occurrence of spaghetti meat was higher in the former compared to the latter (28.9% vs. 7.78%; *P* < 0.001) (Table 5).

### Meat Quality Traits and Chemical Composition of *p.* major

The dietary inclusion of *C. vulgaris* had a significant effect on the color of breast meat: the lightness index decreased (50.7 to 49.6 to 48.9; *P* < 0.01), whereas the red (0.32 to 1.65 to 2.23; *P* < 0.001) and yellow (10.9 to 15.9 to 18.9; *P* < 0.001) indexes significantly increased as the inclusion level of the microalgae increased from 0%, to 3%, and to 6% (Table 6). Also, some textural traits changed, specifically hardness and chewiness (Table 6), whereas proximate composition, lipid and protein oxidation of *p. major* muscle (Table 6), and amino acid and mineral contents (Table 7, Table 8, respectively) were not affected. As for the FA profile (Table 9), the supplementation with the microalgae significantly decreased the rate of arachidic acid (C20:0, −7.14%; *P* ≤ 0.05) in chickens fed diet C3 compared to those fed the control diet. Then, the rates of cis-vaccenic acid (C18:1n7, +5.59%; *P* ≤ 0.05), α-linolenic acid (C18:3n3, + 9.86%; *P* < 0.01), and total n3 FA (+8.59%; *P* ≤ 0.05) increased whereas the n6/n3 ratio decreased (+8.64%; *P* < 0.001) in chickens fed diet C6 compared to those fed the control diet.

**Table 6:** Rheological traits, proximate composition, and lipid and protein oxidation status of *p. major* muscle in chickens fed diets with different inclusion levels of *Chlorella vulgaris* and reared under standard or high room temperature in separated sexes from hatching until commercial slaughtering.

	Experimental diet (D)			Room temperature (T)		Sex (S)		*P* value							RMSE
	C0	C3	C6	Standard	High	Female	Male	D	T	S	D×T	D×S	T×S	D×T×S	
Breasts (n)	36	36	36	54	54	54	54								
pH	5.89	5.90	5.89	5.86	5.92	5.89	5.88	0.908	0.008	0.645	0.854	0.858	0.151	0.480	0.11
L*	50.6 a	49.6^ab^	48.9 b	49.5	49.9	49.6	49.9	0.004	0.411	0.459	0.950	0.862	0.239	0.118	2.12
a*	0.32 a	1.65 b	2.23^c^	1.39	1.41	1.39	1.41	<0.001	0.827	0.879	0.629	0.604	0.124	0.071	0.67
b*	10.9 a	15.9 b	18.9 c	15.4	15.1	15.3	15.2	<0.001	0.467	0.752	0.848	0.530	0.060	0.131	1.90
Hardness (N)	103.0	89.7	99.3	94.3	100.0	97.7	97.0	0.051	0.361	0.882	0.109	0.903	0.943	0.650	23.5
Springiness (mm)	1.61	1.62	1.65	1.60	1.65	1.62	1.63	0.887	0.485	0.847	0.644	0.928	0.485	0.391	0.37
Cohesiveness	0.619	0.605	0.623	0.615	0.616	0.611	0.620	0.398	0.941	0.434	0.668	0.806	0.91	0.336	0.06
Chewiness (N×mm)	98.7	83.0	95.3	84.2	100.0	91.6	91.1	0.070	0.025	0.791	0.040	0.881	0.636	0.503	30.0
Thawing losses (%)	11.9	11.8	12.1	12.5	11.4	11.6	12.3	0.939	0.047	0.183	0.077	0.482	0.910	0.973	2.77
Cooking losses (%)	26.9	26.7	27.2	25.8	28.1	26.7	27.2	0.782	<0.001	0.455	0.161	0.898	0.502	0.694	3.13
Shear force (kg/g)	2.73	2.57	2.58	2.52	2.75	2.62	2.64	0.401	0.253	0.844	0.523	0.846	0.993	0.995	0.56
Breast muscles (n)	24	24	24	36	36	36	36								
Moisture (%)	75.3	75.5	75.3	75.2	75.6	75.3	75.5	0.709	0.122	0.201	0.880	0.887	0.110	0.388	0.90
Ash (%)	1.13	1.13	1.14	1.16	1.11	1.15	1.12	0.518	<0.001	0.030	0.223	0.583	0.911	0.363	0.04
Protein (%)	21.7	21.7	21.8	21.9	21.5	21.9	21.6	0.766	0.056	0.127	0.319	0.922	0.807	0.143	0.79
Fat (%)	1.99	1.68	1.72	1.73	1.86	1.79	1.80	0.250	0.440	0.940	0.360	0.854	0.057	0.272	0.69
TBARS (mg MDA/kg meat)	0.673	0.488	0.495	0.397	0.707	0.529	0.574	0.259	0.076	0.656	0.391	0.511	0.928	0.765	0.42
Carbonyl (nmol/mg protein)	2.37	2.36	2.52	2.30	2.53	2.27	2.56	0.662	0.182	0.085	0.437	0.859	0.743	0.782	0.71

RSME, root mean square error. C0: Control diet (94% basal diet + 6% soybean meal); C3: 94% basal diet + 3% soybean meal + 3% *C. vulgaris*; C6: 94% basal diet + 6% *C. vulgaris*.

a,b,c Different superscripts within a row indicate a significant difference (*P* ≤ 0.05).

**Table 7:** Profiles of amino acids (mg/100 g) in *p. major* muscle of broiler chickens fed diets with different inclusion levels of *Chlorella vulgaris* and reared under standard or high room temperature in separated sexes from hatching until commercial slaughtering.

	Diet (D)			Temperature (T)		Sex (S)		P value							RMSE
	C0	C3	C6	Standard	High	Female	Male	D	T	S	D×T	D×S	T×S	D×T×S	
Breasts (n)	12	12	12	18	18	18	18								
Histidine	940	889	903	943	879	901	920	0.143	<0.001	0.380	0.151	0.814	0.580	0.720	63.3
Hydroxyproline	379	364	400	386	376	376	386	0.145	0.658	0.525	0.036	0.475	0.457	0.220	43.5
Arginine	1140	1144	1168	1188	1113	1147	1154	0.554	0.024	0.778	0.640	0.077	0.501	0.702	66.6
Serine	726	724	744	757	705	728	735	0.366	0.009	0.545	0.595	0.237	0.546	0.750	36.9
Glycine	771	769	779	810	736	755	791	0.887	0.009	0.043	0.318	0.213	0.601	0.570	50.5
Aspartic acid	1533	1556	1564	1669	1433	1535	1567	0.676	<0.001	0.270	0.440	0.195	0.429	0.931	84.2
Glutamic acid	3136	3177	3204	3388	2957	3136	3208	0.657	0.001	0.225	0.443	0.217	0.475	0.935	174
Threonine	765	770	786	813	735	769	779	0.510	0.007	0.505	0.644	0.171	0.485	0.882	43.2
Alanine	942	952	962	1016	887	940	963	0.654	<0.001	0.179	0.503	0.157	0.672	0.879	49.9
Proline	604	605	613	644	571	595	619	0.774	0.002	0.042	0.390	0.208	0.954	0.636	33.7
Lysine	1586	1614	1619	1714	1499	1593	1619	0.713	0.003	0.444	0.314	0.169	0.380	0.896	100
Methionine	488	487	502	507	477	491	493	0.350	0.058	0.782	0.716	0.167	0.474	0.756	26.6
Tyrosine	646	641	666	664	638	649	653	0.173	0.030	0.720	0.511	0.092	0.539	0.704	33.4
Valine	691	691	707	714	681	692	704	0.789	0.094	0.520	0.823	0.080	0.590	0.991	56.4
Cysteine	239	238	241	241	237	237	241	0.881	0.441	0.488	0.652	0.211	0.741	0.803	14.9
Isoleucine	668	671	692	692	662	673	680	0.476	0.099	0.718	0.794	0.063	0.527	0.913	51.3
Leucine	1260	1261	1289	1315	1225	1262	1278	0.561	0.018	0.509	0.710	0.111	0.500	0.865	73.1
Phenylalanine	719	714	741	739	710	722	727	0.237	0.043	0.693	0.483	0.111	0.558	0.670	39.7
Tryptophan	202	205	203	203	205	206	201	0.762	0.566	0.059	0.902	0.311	0.914	0.056	8.62

RSME, root mean square error. C0: Control diet (94% basal diet + 6% soybean meal); C3: 94% basal diet + 3% soybean meal + 3% *C. vulgaris*; C6: 94% basal diet + 6% *C. vulgaris*.

**Table 8:** Mineral composition (mg/100 g) of *pectoralis major* in broiler chickens fed diets with different inclusion levels of *Chlorella vulgaris* and reared under standard or high room temperature in separated sexes from hatching until commercial slaughtering.

	Diet (D)			Temperature (T)		Sex (S)		*P* value							RMSE
	C0	C3	C6	Standard	High	Female	Male	D	T	S	D×T	D×S	T×S	D×T×S	
Breasts (n)	12	12	12	18	18	18	18								
Na	56.6	55.2	56.2	54.4	57.7	54.1	57.9	0.838	0.095	0.060	0.487	0.363	0.321	0.354	5.67
K	366	365	369	370	364	370	364	0.629	0.073	0.111	0.407	0.101	0.073	0.401	10.8
Ca	6.78	6.77	6.72	6.86	6.66	6.78	6.73	0.978	0.670	0.840	0.849	0.394	0.403	0.335	0.71
Mg	29.5	30.0	30.5	29.4	29.6	30.6	29.4	0.185	0.060	0.010	0.631	0.562	0.407	0.076	1.26
P	231	232	234	234	231	236	229	0.496	0.165	0.009	0.624	0.461	0.282	0.340	7.38
Fe	0.408	0.413	0.406	0.403	0.415	0.410	0.408	0.926	0.457	0.868	0.743	0.788	0.929	0.848	0.05
Cu	0.059	0.057	0.059	0.064	0.053	0.058	0.059	0.857	0.099	0.527	0.354	0.849	0.740	0.924	0.01
Zn	0.863^a^	0.827^ab^	0.815^b^	0.835	0.835	0.819	0.851	0.032	0.987	0.030	0.427	0.176	0.013	0.0002	0.04
Mn	0.012	0.011	0.012	0.012	0.011	0.012	0.011	0.234	0.515	0.019	0.539	0.631	0.593	0.052	0.00
S	189	189	192	189	190	192	188	0.304	0.950	0.006	0.921	0.512	0.505	0.096	4.23

RSME, root mean square error. C0: Control diet (94% basal diet + 6% soybean meal); C3: 94% basal diet + 3% soybean meal + 3% *C. vulgaris*; C6: 94% basal diet + 6% *C. vulgaris*.

a,b Different superscripts within a row indicate a significant difference (*P* ≤ 0.05).

**Table 9:** Fatty acid composition (g/100 g FA) of *pectoralis major* broiler chickens fed diets with different inclusion levels of *Chlorella vulgaris* and reared under standard or high room temperature in separated sexes from hatching until commercial slaughtering.

	Diet (D)			Temperature (T)		Sex (S)		*P* value							RMSE
	C0	C3	C6	Standard	High	Female	Male	D	T	S	D×T	D×S	T×S	D×T×S	
Breasts (n)	24	24	24	36	36	36	36								
C14:0	0.553	0.558	0.542	0.540	0.562	0.548	0.554	0.375	0.024	0.564	0.345	0.344	0.678	0.074	0.04
C16:0	22.9	23.1	22.9	22.8	23.1	23.2	22.7	0.111	0.195	0.026	0.079	0.881	0.165	0.004	0.79
C17:0	0.143	0.144	0.151	0.150	0.141	0.146	0.145	0.228	0.034	0.778	0.675	0.951	0.460	0.692	0.02
C18:0	6.62	6.44	6.36	6.62	6.32	6.57	6.38	0.378	0.064	0.221	0.031	0.777	0.099	0.305	0.67
C20:0	0.144^a^	0.129^b^	0.137^ab^	0.136	0.138	0.137	0.137	0.042	0.679	0.977	0.403	0.955	0.060	0.091	0.02
Other SFA	0.588	0.592	0.589	0.586	0.594	0.586	0.593	0.983	0.677	0.707	0.544	0.986	0.090	0.350	0.08
C16:1n7	4.05	4.29	4.37	4.09	4.38	4.16	4.31	0.182	0.051	0.294	0.068	0.589	0.290	0.733	0.61
C16:1n9	0.448	0.446	0.440	0.460	0.429	0.440	0.448	0.744	0.002	0.410	0.198	0.493	0.831	0.023	0.04
C18:1n7	1.61^a^	1.66^ab^	1.70^b^	1.67	1.65	1.70	1.62	0.037	0.635	0.004	0.918	0.940	0.576	0.287	0.12
C18:1n9	33.4	33.0	33.0	33.2	33.0	33.5	32.8	0.385	0.496	0.022	0.560	0.565	0.364	0.005	1.17
Other MUFA	0.599	0.675	0.723	0.650	0.681	0.673	0.659	<0.001	0.092	0.427	0.299	0.663	0.939	0.479	0.08
C18:2n6	25.0	24.9	24.9	25.0	24.9	24.5	25.5	0.960	0.961	0.007	0.232	0.701	0.706	0.010	1.51
C18:3n6	0.287	0.268	0.279	0.278	0.278	0.270	0.286	0.159	0.940	0.061	0.233	0.206	0.343	0.392	0.03
C20:2n6	0.228	0.218	0.234	0.218	0.236	0.219	0.235	0.615	0.236	0.220	0.956	0.731	0.381	0.307	0.06
C20:4n6	0.618	0.629	0.596	0.656	0.573	0.598	0.632	0.681	0.046	0.273	0.674	0.787	0.286	0.086	0.13
C22:4n6	0.179	0.182	0.177	0.190	0.169	0.184	0.175	0.966	0.494	0.623	0.889	0.793	0.645	0.687	0.08
C18:3n3	2.13^a^	2.24^ab^	2.34^b^	2.18	2.29	2.19	2.29	0.005	0.043	0.052	0.032	0.691	0.449	0.003	0.21
C20:5n3 (EPA)	0.079	0.063	0.075	0.050	0.094	0.072	0.072	0.512	0.0004	0.961	0.563	0.804	0.920	0.631	0.05
C22:5n3	0.159	0.175	0.177	0.183	0.158	0.162	0.179	0.264	0.013	0.096	0.140	0.948	0.521	0.614	0.04
C22:6n3 (DHA)	0.128	0.130	0.142	0.132	0.135	0.131	0.136	0.340	0.709	0.502	0.080	0.813	0.667	0.258	0.03
Other PUFA	0.080	0.067	0.073	0.071	0.076	0.069	0.077	0.421	0.544	0.352	0.010	0.747	0.950	0.835	0.03
SFA	30.9	30.9	30.7	30.9	30.8	31.2	30.6	0.781	0.920	0.041	0.032	0.792	0.056	0.013	1.23
MUFA	40.1	40.1	40.3	40.1	40.2	40.4	39.9	0.935	0.919	0.131	0.827	0.796	0.268	0.038	1.61
PUFA	28.9	29.0	29.0	29.0	29.0	28.4	29.6	0.986	0.982	0.007	0.154	0.743	0.738	0.006	1.80
PUFA/SFA	0.938	0.941	0.947	0.941	0.943	0.915	0.970	0.926	0.909	0.007	0.050	0.837	0.283	0.005	0.08
n6	26.4	26.3	26.2	26.4	26.2	25.8	26.8	0.955	0.669	0.006	0.232	0.777	0.768	0.008	1.59
n3	2.56^a^	2.66^ab^	2.78^b^	2.59	2.74	2.60	2.63	0.028	0.027	0.062	0.018	0.617	0.624	0.009	0.28
n6/n3	10.4^a^	9.9^ab^	9.5^b^	10.2	9.6	10.0	9.9	0.0004	0.0008	0.598	0.012	0.659	0.700	0.092	0.73

RSME, root mean square error. SFA: saturated fatty acids; MUFA: monounsaturated fatty acids; PUFA: polyunsaturated fatty acids. C0: Control diet (94% basal diet + 6% soybean meal); C3: 94% basal diet + 3% soybean meal + 3% *C. vulgaris*; C6: 94% basal diet + 6% *C. vulgaris*.

a,b,c Different superscripts within a row indicate a significant difference (*P* ≤ 0.05).

Exposure of broiler chickens to chronic heat stress resulted in a higher meat pH (5.86 *vs*. 5.92; *P* < 0.01), decreased thawing losses (12.5% vs. 11.4%; *P* = 0.05), and increased cooking losses (25.8% vs. 28.1%; *P* < 0.001) compared to standard temperature conditions (Table 6). Meat chewiness was significantly higher (*P* < 0.01) in heat-stressed chickens compared with those reared under standard conditions, whereas no significant difference in TBARS level and carbonyls content in *p. major* muscle was found between chickens kept at the 2 different temperature conditions (Table 6). As for the chemical composition, a chronic heat stress increased the rate of myristic acid (C14:0, +3.57%; *P* ≤ 0.05), α-linolenic acid (C18:3n3, +4.80%; *P* ≤ 0.05), eicosapentaenoic acid (C20:5n3, +44.4%; *P* < 0.001), and total n3 FA (+5.47%; *P* ≤ 0.05), which was associated to a decreased ratio of palmitoleic (C16:1n9, −6.98%; *P* < 0.01), arachidonic (C20:4n6, −15.8%; *P* ≤ 0.05), and docosapentaenoic (C22:5n3, −15.5%; *P* = 0.01) acids (Table 9). Exposure to chronic heat stress significantly decreased the content of several amino acids (from −4.07% to −16.47%) (Table 7), namely histidine, arginine, serine, glycine, aspartic acid, glutamic acid, threonine, alanine, proline, lysine, tyrosine, leucine, and phenylalanine in broiler breast meat.

As for differences according to sex, the breast meat of females had a higher rate of palmitic acid (C16:0, +1.85%; *P* ≤ 0.05), total SFA (+1.92%; *P* ≤ 0.05), vaccenic (C18:1n7, +4.71%; *P* < 0.01), and oleic (C18:1n9*,* +1.94%; *P* ≤ 0.05) acids in comparison to males, whereas the rates of linoleic acid (C18:2n6, −4.09%; *P* = 0.06), γ-linoleic acid (C18:3n6, −7.41%; *P* < 0.01), total n6 FA (−4.11%; *P* < 0.01), and total PUFA (−4.19%; *P* < 0.01) were lower (Table 9). Then, females had lower contents of hydroxyproline (−2.66%; *P* ≤ 0.05) and proline (−4.03%; *P* ≤ 0.05) (Table 7). Despite significant, differences in mineral composition (higher contents of magnesium, phosphorus, manganese, and sulfur, and lower content of zinc; *P* ≤ 0.05) between breast meat of females and males were in a narrow range from a numerical point of view (Table 8).

### Sensory Attributes of Cooked Breasts

Most sensory attributes of meat from chickens fed diets with different inclusion levels of the microalgae were not differentiated by the trained panelists (Table 10). Namely, the panelists only found that the *C. vulgaris* inclusion significantly affected the meat color (*P* < 0.001), which intensity increased with the microalgae inclusion level, and the sweet taste, less intense in the meat from chickens fed diet C6 compared to those fed the control diet (*P* ≤ 0.05). Moreover, the brothy flavor intensity differed between the meat of chickens fed diet C3 or diet C6 (*P* ≤ 0.05), with meat from chickens fed diet C0 showing intermediate values.

**Table 10:** Score for sensory attributes of *pectoralis major* in broiler chickens fed diets with different inclusion levels of *Chlorella vulgaris* and reared under standard or high room temperature in separated sexes from hatching until commercial slaughtering.

	Diet (D)			Temperature (T)		Sex (S)		*P* value							RMSE
	C0	C3	C6	Standard	High	Female	Male	D	T	S	D×T	D×S	T×S	D×T×S	
Color	4.42^a^	5.19^b^	5.70^c^	5.10	5.11	5.10	5.11	<0.001	0.905	0.960	0.716	0.316	0.559	0.137	0.25
Flavor															
Brothy	4.26^ab^	3.93^a^	4.30^b^	3.93	4.39	4.29	4.03	0.029	0.0003	0.043	0.636	0.862	0.720	0.659	0.31
Meaty/umami	5.71	5.37	5.46	5.38	5.66	5.61	5.42	0.064	0.021	0.104	0.516	0.024	0.603	0.016	0.30
Salty	3.36	3.44	3.36	3.29	3.49	3.42	3.36	0.816	0.080	0.596	0.528	0.042	0.173	0.187	0.29
Sweet	4.20^a^	4.02^ab^	3.82^b^	3.50	4.50	4.17	3.86	0.022	<0.001	0.006	0.516	0.274	0.995	0.0091	0.28
Pleasantness	5.69	5.65	5.81	5.57	5.86	5.66	5.78	0.803	0.137	0.545	0.677	0.757	0.593	0.737	0.49
Abnormal taste	0.433	0.417	0.375	0.459	0.357	0.401	0.415	0.851	0.241	0.872	0.234	0.873	0.254	0.248	0.22
Abnormal smell	0.367	0.351	0.293	0.377	0.296	0.345	0.328	0.734	0.320	0.837	0.211	0.147	0.482	0.524	0.20
Texture															
Hardness	3.05	3.28	2.85	3.17	2.96	3.10	3.03	0.086	0.178	0.664	0.633	0.299	0.046	0.749	0.39
Juiciness	4.01	4.27	4.23	4.17	4.23	4.27	4.13	0.645	0.729	0.406	0.634	0.727	0.685	0.059	0.41
Chewiness	3.95	3.98	4.16	4.17	3.89	4.04	4.02	0.565	0.101	0.903	0.254	0.253	0.389	0.661	0.43
Cohesiveness	3.07	3.24	3.27	3.35	3.02	3.26	3.11	0.601	0.056	0.395	0.562	0.634	0.684	0.810	0.43
Fibrosity	4.70	4.66	4.76	4.92	4.49	4.61	4.80	0.868	0.006	0.204	0.116	0.182	0.041	0.448	0.38

RSME, root mean square error. C0: Control diet (94% basal diet + 6% soybean meal); C3: 94% basal diet + 3% soybean meal + 3% *C. vulgaris*; C6: 94% basal diet + 6% *C. vulgaris*.

a,b Different superscripts within a row indicate a significant difference (*P* ≤ 0.05).

Then, the meat from the birds exposed to chronic heat stress had more intense brothy (*P* < 0.001), meaty (*P* ≤ 0.05), salty (*P* < 0.10), and sweet (*P* < 0.001) flavor, and was less fibrous (*P* < 0.01) compared to the meat from the birds reared under standard conditions.

Finally, the meat from females displayed more intense brothy (*P* ≤ 0.05) and sweet (*P* < 0.01) flavors compared to their male counterparts.

## DISCUSSION

### Effect of the Dietary Inclusion of C. vulgaris

The dietary inclusion of microalgae replacing the current crop protein sources, such as soybean meal, has been proposed to improve sustainability and reduce environmental impacts of animal production (Almomani et al., 2023). To this purpose, different microalgae (*Chlorella* spp., *Arthrospira platensis*/Spirulina, *Schizochytrium* spp.) have been tested in broiler chicken production (Madeira et al., 2017; Alfaia et al., 2021; Boskovic Cabrol et al., 2022b). Decrease of feed intake and consequently growth found in the present study at the highest inclusion level (6%) of the microalgae could be due to decreased diet palatability because of the characteristic algal odor (Kang et al., 2013; Abdelnour et al., 2019). On the other hand, the birds fed 3% *C. vulgaris* had the highest numerical feed intake and body weight, suggesting that the effect of microalgae on palatability could be dose-dependent. Depression in performance following the highest inclusion levels of *C. vulgaris* could also be due to an increased digesta viscosity at the gut level because of the gelation of microalgae proteins and the high non-starch polysaccharide contents of microalgae (Evans et al., 2015; Alfaia et al., 2021; Boskovic Cabrol et al., 2022b) which can negatively impact on diet digestibility. The few available studies about the use of *C. vulgaris* have reported decreased growth performance and feed intake at dietary inclusion levels equal or higher than 10% (Boskovic Cabrol et al., 2022b).

In the present study, dietary microalgae inclusion did not alleviate the negative impact of heat stress on broiler performances. Contrarily, beneficial effects of dietary microalgae or carotenoids from microalgae supplementation on performance and health of poultry exposed to heat stress (Ziar-Larimi et al., 2018; Tolba et al., 2020) where results in the different studies can also depend on differences in management of animals, besides type and level of microalgae. In fact, Tolba et al. (2020) reported that a dietary supplementation with microalgae (*Hematococcus pluvialis*) astaxanthin (10–80 mg/kg) to broilers exposed to heat stress linearly decreased hepatic mRNA levels of several redox status-controlling genes, heat shock protein 70 (**HSP70**), heat shock transcription factor 1 (**HSTF1**), c-Jun N-terminal kinase 1 (**JNK1**), tumor necrosis factor-α, and sterol regulatory element-binding protein 1 (**SREBP1**), and increased diacylglycerol acyltransferase 2 (**DGAT2**) mRNA levels, modulating molecular profiles of stress, inflammation, and lipid metabolism. Moreover, the dietary supplementation with very low levels of *C. vulgaris* (0.2 g/kg) has been reported to improve growth performance in heat-stress broilers (Ziar-Larimi et al., 2018). The supplementation in drinking water (300–500 mg/kg) with the same microalgae had beneficial impacts on serum contents of triglycerides, cholesterol, LDL, and HDL in laying hens exposed to chronic heat stress (Moradi kor et al., 2016), which were ascribed to a radical scavenging and hypolipidemic action of the β-1, 3-glucan found in the cell wall of this microalgae and/or the presence of antioxidant substances.

In our trial, as expected, differences in growth performance according to the dietary inclusion level of *C. vulgaris* accounted for differences in carcass traits, where the lighter chickens fed the diet with the highest inclusion level of *C. vulgaris* showed greater breast and *p. major* yields compared to the other chickens (Boskovic Cabrol et al., 2022b). On the contrary, in a previous study (Gatrell et al., 2018), a moderate dietary inclusion (4–8%) of a defatted green microalga (*Nannochloropsis oceanica*) had been found to increase breast muscle protein expression of biomarkers of protein synthesis regulation, in particular, eukaryotic translation initiation factor 4E (**elf4E**), S6 ribosomal protein (**S6**), and mammalian target of rapamycin (**mTOR**).

If previous and present results outlined that even a moderate replacement (6%) of conventional protein sources, such as soybean, with microalgae is not feasible without negative consequences on performance of broiler chickens, lower inclusion levels (until 3%) could have positive effects on meat quality due to the bioactive compounds with different functional roles that these microalgae can supply.

With respect to meat quality and consistently with previous reports (El-Bahr et al., 2020), the present study confirmed that the dietary inclusion of *C. vulgaris* did not affect meat technological traits, whereas Boskovic Cabrol et al. (2022b) had found that highest inclusion rate (20%) increased meat water holding capacity in raw meat and decreased cooking losses. Then, the xanthophyll content of *C. vulgaris*, in particular lutein (Kulkarni and Nikolov, 2018), accounted for the substantial differences we measured in meat color, even at the lowest inclusion rate (3%). Consistently with our results, earlier researches reported decreased lightness and increased redness and yellowness indexes measured on the breast meat of chickens fed diets containing *C. vulgaris* compared to chickens fed a control diet (Alfaia et al., 2021; Boskovic Cabrol et al., 2022b). In our trial, color differences were confirmed even in the cooked meat by the trained panelists at the sensory evaluation. According to Boskovic Cabrol et al. (2022b), a 10% dietary inclusion of *C. vulgaris* was associated to the most appropriate meat color for consumers preferring yellow meat color. On the other hand, a very low inclusion level of the same microalgae (0.05% to 0.5%) has been found to be insufficient to change meat color to an appreciable extent (An et al., 2016).

As for the nutritional quality, proximate composition of breast meat is not likely to be affected by the dietary inclusion of *C. vulgaris*, whereas its FA profile is expected to change according to that of the microalgae used and according to the dietary inclusion level. In the present trial, consistently with previous reports (Spínola et al., 2023), α-linolenic acid (18:3n3) was the predominant FA in *C. vulgaris*, which accounted for its significantly higher proportion, and for the significantly higher n3 PUFA rate, in the breast meat of broiler chickens fed the diets with the highest inclusion level of *C. vulgaris* compared to the control diet. Nevertheless, Boskovic Cabrol et al. (2022b) reported a linear increase in all n3 PUFA, including EPA and DHA, when *C. vulgaris* was incorporated at 10, 15, and 20% in broilers diets which was not confirmed in the present study in which lower microalgae inclusion levels with a low-fat content (<1%) were used compared to the same study. We neither recorded any protective effect of dietary *C. vulgaris* inclusion against lipid oxidation in breast meat, as measured by the TBARs values, consistently with previous results (Alfaia et al., 2021; Boskovic Cabrol et al., 2022b).

As for minerals, we can expect that large differences in mineral composition of the diets according to the inclusion level of microalgae could change the mineral composition of breast meat in broiler chickens (Stef and Gergen, 2012). Indeed, in our trial, the contents of several minerals changed according to the dietary inclusion level of *C. vulgaris*, but differences were in a narrow range from a numerical point of view and not so high to be associated to substantial changes in meat nutritional value for these nutrients. Contrarily, Boskovic Cabrol et al. (2022a) reported an increase in K, Ca, Mg, P, and Fe content in raw breast meat when soybean meal was replaced with 15-20% of *C. vulgaris* in the broilers diet, whereas Costa et al. (2022) found an increase in Ba and I content with diets including 15% of brown macroalgae (*Laminaria digitata*). These results suggest that the dietary effect of algae on meat mineral composition is dose dependent.

As for amino acids, in the present trial the microalgae inclusion level did not significantly affect the contents of the different amino acids, as found by El-Bahr et al. (2020). Contrarily, Boskovic Cabrol et al. (2022a) found that the inclusion of *C. vulgaris* at higher rates (10-20%) could increase the proportions of arginine and threonine and decrease that of lysine and cysteine, where amino acid composition can affect meat sensorial properties. Indeed, in the present trial, the 6% dietary microalgae inclusion decreased the sweet flavor of cooked meat. In fact, sweet taste is determined by the content of alanine, glycine, proline, serine, and threonine, while the bitter taste is related to the content of histidine, isoleucine, leucine, methionine, phenylalanine, tryptophan, and valine (Pérez-Santaescolástica et al., 2018). While it is worth noting that the taste is determined by free amino acids, which were not analyzed in the present study, previous studies reported an increase in umami and chicken flavor of meat from broiler chickens fed diets in which Spirulina replaced 50% of soybean meal (Altmann et al., 2020).

Regarding growth-related breast myopathies, we did not find a significant effect of the dietary inclusion of microalgae on the reduction of occurrence of these conditions. The decreased WB occurrence (at a level approaching significance) in chickens fed C6 diet compared to those fed the other diets has to be attributed to the lower growth rate and breast weight of the former chickens compared to the latter ones (Bordignon et al., 2022) rather than to a possible functional role played by the microalgae. Khan et al. (2021) reported only a numerical decrease in WS occurrence when chickens were fed diets containing 2% of DHA-rich microalgae. Mullenix et al. (2022) reported no effect on WB myopathy scores when *Spirulina* replaced until 50% of soybean meal in the diet formulation.

### Effect of Heat Stress

In our trial, exposure to heat stress reduced feed intake and therefore lowered daily weight gain. In poultry, exposure to heat is known to reduce feed intake to favor bird thermoregulation by lowering metabolic heat production (Gous and Morris, 2005). For birds approaching market age, it is challenging to maintain thermal homeostasis due to the large body mass and high metabolism rate associated with the rapid growth of commercial crossbred genotypes (Borges et al., 2003), which corroborates our findings that changes related to heat stress were most evident during the finisher phase. In fact, in our trial, differences in daily weight gain and feed intake between chickens kept in the 2 rooms started to appear during the second period, but became relevant and significant during the third and last period of growth. The weak effects observed during the second period can be ascribed both to the higher tolerance of chickens at this age towards higher temperatures compared to the third period, both to the fact that the diets were in a mash form which is known to reduce feed intake compared to a crumble or pelleted diet (Jafarnejad et al., 2010; El-Bahr et al., 2020). However, these changes did not have repercussions on the final body weight in the present study. Several studies reported a significant negative impact of heat stress on performance of broiler chickens (Lu et al., 2019; Zampiga et al., 2021b; Huerta et al., 2023). High environmental temperatures activate the hypothalamic-pituitary-adrenal axis, resulting in increased levels of corticosterone and impaired intestinal barrier stability and integrity which can facilitate infections and/or hypoxia (Quinteiro-Filho et al., 2012; Brugaletta et al., 2020; Tabler et al., 2020), besides inflammatory processes that can cause crypt hyperplasia and villus atrophy (He et al., 2018; Liu et al., 2020) and, thus, lower utilization of nutrients. Although the effect of feed intake on body weight is undisputable, the alteration of growth performances is partly caused by increased respiratory rate, followed by glycogen depletion, and a negative energy balance (He et al., 2018).

As for carcass traits, results from the present study showed a reduction of breast yield and an increase of thigh proportions in chickens exposed to chronic heat stress associated to lower body weight. Reduced breast yield, also previously reported by other authors (Oliveira et al., 2006; Zhang et al., 2012; Lu et al., 2019; El-Tarabany et al., 2021) in broiler chickens exposed to chronic heat stress, is likely a consequence of protein catabolism resulting in decreased amino acid content in the muscles from broilers kept at high temperatures, as found herein.

In fact, in the present study, chronic heat stress triggered some changes in the amino acid composition of the *p. major* muscle, causing a decrease of the contents of most amino acid, even if total protein content was not affected. As reported in previous studies in heat-stressed broiler chickens, chronic heat stress increases circulating corticosterone levels, suppressing muscle protein synthesis while increasing catabolism to provide amino acids as metabolic substrates to supply energy by liver gluconeogenesis (El-Tarabany et al., 2021; Ma et al., 2021; Zampiga et al., 2021b).

Then, as for meat quality, chronic heat stress also leads to an exhaustion of muscle glycogen reserves in muscle*,* causing higher meat pH (Zeferino et al., 2016) which is associate to a higher protein functionality and capacity to bind water, and, thus, lower thawing losses, as found herein in meat from broilers kept under chronic heat stress conditions. On the other hand, higher cooking losses in heat-stressed birds were measured by both Zeferino et al. (2016) and the present trial, probably due to a more pronounced protein denaturation during cooking that would reduce its ability to bind water (Zhang et al., 2012). Although this was not confirmed by statistical analysis, numerical differences in hardness among breasts from broilers exposed to different temperatures were recorded, resulting in significantly higher chewiness of breast meat from broilers kept under heat stress than those under thermoneutral conditions. Nevertheless, trained panelists did not determine the effect of the temperature on chewiness.

Moreover, the temperature did not have effect on mineral composition and vaguely influenced chemical composition. As for the meat proximate composition, results among studies about the effect of heat stress are not consistent: some authors found decreased meat protein content and increased fat content in heat stressed chickens (Rosa et al., 2007), whereas others did not find differences (Huerta et al., 2023; present study). On the other hand, chronic heat exposure can alter muscle fatty acid profiles, with increased SFA rate and reduced MUFA and PUFA rates in chicken breasts (Tavaniello et al., 2020; El-Tarabany et al., 2021). Nevertheless, under our conditions, total SFA, MUFA, and PUFA rates did not change, but meat from heat stressed bird had higher n3 PUFA content. A significant increase in n3 PUFA and myristic acid in birds exposed to heat stress was also reported by other authors (Tavaniello et al., 2020; Salah et al., 2021). Due to the deterioration of PUFA upon initiation of oxidative stress, the DHA content was lower in the *p. major* muscle of broilers exposed to heat stress compared to those kept at thermoneutral conditions. However, in line with previous findings (Salah et al., 2021), the levels of α-linolenic acid in the breast remain stable in heat-stressed broilers. Moreover, the same oxidation level was recorded in muscles from broilers kept under standard and chronic heat-stress conditions. Indeed, previous studies (Wang et al., 2009; Azad et al., 2010; Habibian et al., 2016) reported that exposure to acute and chronic heat stress resulted in a 1.2-to-4-fold increase of MDA in the breast meat of chickens due to oxidative stress and accumulation of free radicals; this is consistent with the trend to increased MDA (+45.7%; *P* < 0.10) we found in the present study in muscles from broilers exposed to chronic heat stress. In addition, the aforementioned studies also emphasized that the degree of lipid oxidation in breast meat was lower in chickens exposed to chronic heat stress compared with those exposed to an acute stress.

While changes in fatty acid and amino acid composition, besides in lipid oxidation, could affect meat flavor to a certain extent, in the present study trained panelists reported increased intensity of brothy, meaty, salty, and sweet flavor in meat of chickens exposed to heat stress compared to meat from the control group. Indeed, the differences in amino acid contents and the higher cooking losses could have decreased the taste/flavor intensity in meat from broilers exposed to chronic heat stress compared to the other chickens, as previously reported in meat with higher cooking loss (Hopkins et al., 2006). Nevertheless, Sandercock et al. (2001) reported that acute heat stress did not impact on eating quality in 35-D-old birds, whereas flavor intensity of meat decreased in heat-stressed 63-D-old birds. On the other hand, a complete analysis of flavor volatile compounds would be required to obtain a comprehensive overview about the effect of heat stress on meat flavor.

Finally, no differences in myopathies occurrence were observed between temperature treatments, although the overall rate of defective muscles was higher in birds exposed to heat stress (56.67%) than in those kept at thermoneutral conditions (48.89%). Micro ischemia caused by capillary support reduction in the *p. major* muscle in broiler chickens exposed to high environmental temperatures (Joiner et al., 2014) or oxidative stress could account for this result.

A recent study showed that the severity of WS myopathy increased in broiler chickens exposed to chronic heat stress (32 ºC from d 21 onwards), despite the lower body weight gain of those broilers (Aslam et al., 2021). Authors suggested that chronic heat stress related hypoxia and increased corticosterone levels, resulting in increased protein degradation, were likely responsible for increasing the WS severity in broilers subjected to heat stress. This issue is worth of further investigations in view of the enormous economic losses caused by heat stress and myopathy occurrence to the poultry industry (He et al., 2018; Barbut, 2020; Zhang et al., 2020).

### The Effect of Sex

In broiler chickens, sexual dimorphism in relation to growth traits, as measured in the present study, has been previously well-documented (Trocino et al., 2015; Cygan-Szczegielniak et al., 2019; Pascual et al., 2020) and has been attributed to phenotypic and transcriptional differences between sexes linked to feed efficiency in intestinal and muscular tissues (Reyer et al., 2018). As for slaughter results and carcass traits, the present study reported greater breast and *p. major* muscle yields and lower legs yield in females than males, corroborating previous findings (López et al., 2011; Huerta et al., 2023), whereas most meat quality traits did not differ between sexes. Nevertheless, we observed sex-specific differences in fatty acid composition between females and males, as described in previous studies (Domínguez et al., 2014), caused by differences in metabolism. In details, higher proportion of PUFA in males compared with females due to higher rates of linoleic and γ-linolenic acid are in line with previous reports in both fast- (Stanišić et al., 2023) and slow-growing chickens (Cerolini et al., 2019; Bongiorno et al. 2022). The higher total SFA and palmitic acid rates in females compared with males (Baéza et al., 2010; Stanišić et al., 2023; present study) have been attributed to their higher fat deposition in peripheral tissues. Dridi et al. (2007) also reported significantly higher SCD gene expression in the kidney breast muscle, proventriculus, and intestine of female compared with male chickens, where this gene encodes Δ9-desaturase, a central enzyme involved in the long-chain monounsaturated fatty acids (**LC-MUFA**) from long-chain saturated fatty acids (**LC-SFA**), which catalyze stearic to oleic acid (higher in females than in males in the present study).

Regarding sensory attributes, the higher intensity of brothy and sweet flavor in meat in male compared with that of female chickens could be due to the previously discussed differences in amino acid, mineral and fatty acid composition. Nevertheless, Stęczny and Kokoszynski (2019) did not find any differences in meat sensory attributes between females and males in 42-D-old Ross 308 broiler chickens, whereas Hussein et al. (2019) reported that females had tender breast meat than males.

As for myopathies, in the present trial as in previous ones (Castilho et al., 2021; Bordignon et al., 2022), WS occurrence was similar in the 2 sexes, whereas WB occurrence was higher in males compared to female birds and SM showed an opposite trend (Trocino et al., 2015; Bordignon et al., 2022; Novoa et al., 2022) which remain worth of further investigation at a molecular level to understand why and how the 2 myopathies differently develop in the 2 sexes.

## CONCLUSIONS

Based on growth performance, carcass, and meat quality traits, the microalgae *C. vulgaris* can be used to replace until 3% of soybean meal in diets for broiler chickens without negative effects, where microalgae are associated to a lower environmental impact compared to conventional raw materials. The microalgae dietary inclusion also positively affected breast meat color according to consumers’ preferences, whereas more investigation would be necessary to elucidate the effects on meat sensory properties. On the other hand, the improvement of the meat fatty acid profile was quite weak due to the low-fat content of the *C. vulgaris* we used. Finally, under our conditions, the inclusion of the microalgae did not mitigate the negative effects of a chronic heat stress on growth performance nor reduced the occurrence of any of the most recent myopathies, i.e. white striping, wooden breast, and spaghetti meat, which has to be confirmed under more extreme thermal conditions and with higher inclusion levels of the microalgae.

## DISCLOSURES

The authors declare no conflicts of interest.
